# Multiphysics Simulation of Shell Solidification Evolution in CSP Thin Slab Casting of Silicon Steel with Box-Type Electromagnetic Stirring

**DOI:** 10.3390/ma19122521

**Published:** 2026-06-11

**Authors:** Hong Xiao, Jian Liu, Lang Wang, Sheng-Zhao Wang, Yan-Zhong Li, Pu Wang

**Affiliations:** 1 Magnetoelectric Research Institute, Hunan Zhongke Electric Co., Ltd., Yueyang 414000, China; 2 Jingye Steel Group Co., Ltd., Shijiazhuang 050400, China; 3 School of Physics and Materials Science, Nanchang University, Nanchang 330031, China

**Keywords:** slab continuous casting, shell solidification evolution, box-type electromagnetic stirrer (B-EMS), silicon steel, equiaxed grain ratio

## Abstract

**Highlights:**

- B-EMS is innovatively applied to the high-speed CSP thin strip continuous casting production line, which solves the bottleneck of loading electromagnetic field on CSP production lines and effectively reduces the columnar grain ratio in the slabs.
- Multiphysics simulation reveals asymmetric electromagnetic forces generate unique width-directional flow patterns, accelerating superheat dissipation and intensifying solidification front scouring.
- Within the optimal current range, the solidified shell thickness increases by 2–3%.
- Industrial trials achieve a 30% increase in equiaxed grain ratio at 800 A, offering a practical solution for anisotropy-related defects.

**Abstract:**

In CSP thin slab casting, high casting speeds promote excessive columnar grain growth, leading to low equiaxed grain ratios in non-oriented silicon steel and resulting in wrinkling defects. This study employs a box-type electromagnetic stirrer (B-EMS) to address this issue. A multiphysics model was established, in which grain transformation and its associated effects were neglected. The effects of B-EMS on the flow of molten steel, temperature distribution and evolution of solidified shell were analyzed, and industrial trials were conducted to verify the influence of B-EMS on grains. Results show that B-EMS generates asymmetric magnetic fields and electromagnetic forces, driving width-directional flow that enhances scouring of the solidification front. Compared with the experiment and simulation, the error in the magnetic field excited by B-EMS is within 5%. Under 800 A current, narrow-face center shell thickness increased from 22.88 mm (no stirring) to 23.62 mm (starting side) and 23.21 mm (pushing side). The central mushy zone area and liquid fraction decreased significantly, indicating accelerated solidification and more uniform shell growth. Industrial trials confirmed that the equiaxed grain ratio increased to approximately 30%, with significantly improved internal strand quality. This study demonstrates B-EMS’s metallurgical effects in regulating solidification structure, optimizing shell morphology, and improving continuous casting slab quality. The numerical simulation can be correlated with the industrial production process to better guide manufacturing practices.

## 1. Introduction

For silicon steel, a key soft magnetic material used in power systems and electric vehicle drive motors, the final performance critically depends on the solidification microstructure formed during continuous casting [1]. The slab’s solidified structure is generally categorized into three zones: the surface chill layer, the columnar grain region, and the central equiaxed grain region. The primary role of the surface chill zone is to ensure production safety (preventing breakouts), but its contribution to magnetic properties is limited. Columnar grain structures, especially coarse ones, are significantly detrimental: first, they cause magnetic anisotropy, affecting the uniformity of magnetic induction; secondly, they easily lead to defects such as cracks and warping during hot rolling due to stress concentration, reducing yield [2,3,4,5]. In contrast, a fine and uniform equiaxed grain structure is ideal. It can restrain the excessive growth of columnar grains, reduce central defects, and impart excellent isotropic magnetic properties to silicon steel. Simultaneously, due to its high grain boundary strength and good plasticity, this structure can significantly enhance the hot workability of silicon steel, thereby ensuring stable production and product quality.

In current industrial production, methods such as controlling casting superheat and optimizing secondary cooling water distribution have limited effectiveness in increasing the equiaxed grain ratio [6,7,8,9]. Traveling magnetic field electromagnetic stirring (S-EMS) technology, which induces forced convection in the molten steel to dissipate superheat and fragment dendrites, has become an effective means for controlling the solidification structure [10,11,12,13]. Studies show that roller-type electromagnetic stirring (R-EMS) can generate high-intensity vortex flow (velocity > 0.3 m/s) in the secondary cooling zone, increasing the equiaxed grain ratio from 16% to 36% [14]. The use of dual R-EMS units intensifies molten steel flow near the solidification front, promoting superheat dissipation and raising the slab’s central equiaxed grain ratio to 69% [15]. In a related study, Xiao et al. [16]. investigated the application of S-EMS in the continuous casting of PW800 silicon steel, demonstrating that this technology effectively promotes the columnar-to-equiaxed transition (CET) by generating intense eddy currents near the solidification front, significantly increasing the average equiaxed grain ratio from 8% to 33%. In contrast, box-type electromagnetic stirring (B-EMS), installed behind the roll gap, offers a larger operational space. B-EMS induces macroscopic convection with relatively low flow velocities but on a broader spatial scale within the strand, making it particularly suitable for wide slabs exceeding 1500 mm in width. Its primary advantage lies in promoting superheat dissipation and achieving uniform temperature distribution in the liquid pool. From the perspective of operating mechanisms, these two techniques have distinct focuses: S-EMS demonstrates significant efficacy in grain refinement and increasing the equiaxed grain ratio through intense localized stirring near the solidification front, although with a relatively limited effective range. B-EMS, on the other hand, concentrates on macroscopic heat transfer and fluid flow across the entire liquid pool, more effectively homogenizing superheat and providing a stable temperature field for large format slabs, though with a weaker direct impact on the solidification front.

Due to the large width-to-thickness ratio of wide slabs and the complex equipment layout in the secondary cooling zone, designing and installing roll-type electromagnetic stirrers between rolls presents considerable technical challenges. Currently, only a few companies worldwide can provide complete equipment solutions, such as Europe’s ABB and Rotelec, and China’s Hunan Zhongke Electric Co., Ltd. Based on differences in coil design and installation methods, the main types currently available on the market include Nippon Steel’s DKS insertable inter roll stirrer, ABB’s roll rear box-type stirrer, and Rotelec’s roll-type stirrer.

Regarding the influence of electromagnetic stirrer configuration on metallurgical effects, some research progress has been made. Gong et al. [17] pointed out that using paired side-by-side roll-type electromagnetic stirrers can generate stronger electromagnetic forces. Furthermore, when two pairs of stirrers are arranged separately with opposing electromagnetic forces, it promotes molten steel flow more effectively compared to same-direction application, thereby enhancing the equiaxed grain ratio. Lei et al. [18] analyzed the flow and solidification behavior under roll-type stirring, revealing that the induced molten steel flow field exhibits a butterfly shaped distribution, and that the independent installation of upper and lower stirrer pairs contributes to optimizing the internal flow field, significantly improving slab quality. In thin slab continuous casting applications, Wang et al. [19] found that adopting an alternating operation mode of roll-type electromagnetic stirring (S-EMS) can form multiple vortex structures within the slab. The backflow of molten steel effectively promotes equiaxed grain formation, increasing the equiaxed grain ratio by 26%.

Compared to roll-type stirring, box-type electromagnetic stirrers are installed behind the roll array without requiring major modifications to the segment or rolls, offering significant advantages in engineering applications. Research has shown that this technology can effectively control the solidification process: on one hand, the generated magnetic field exhibits an asymmetric distribution between the inner and outer arcs of the slab [20]; on the other hand, even under unfavorable conditions with high superheat, it can increase the equiaxed grain ratio of silicon steel to over 50%, effectively offsetting the negative effects of elevated superheat [21].

However, the application of box-type electromagnetic stirrers (B-EMSs) in CSP thin slab casting of silicon steel has rarely been reported, and systematic analysis of the characteristics of their traveling magnetic fields and the influence of magnetohydrodynamic (MHD) mechanisms on stirring behavior in the secondary cooling zone remains lacking. Existing research has primarily focused on roller-type electromagnetic stirring technology, while the unique asymmetric electromagnetic force distribution of B-EMS and its induced flow patterns along the width direction are not well understood. To address this research gap, this paper establishes a segmented multi-field coupling model encompassing electromagnetic, flow, heat transfer, and solidification phenomena. The mechanism by which the traveling magnetic field, under different current parameters, affects metallurgical transport and the evolution of the solidification structure in the secondary cooling zone of silicon steel slabs was revealed, and the reliability of the model was verified through industrial experiments. This work provides a theoretical basis and guidance for optimizing B-EMS process parameters and improving the internal quality of the slab.

## 2. Model Establishment

**Figure 1:**
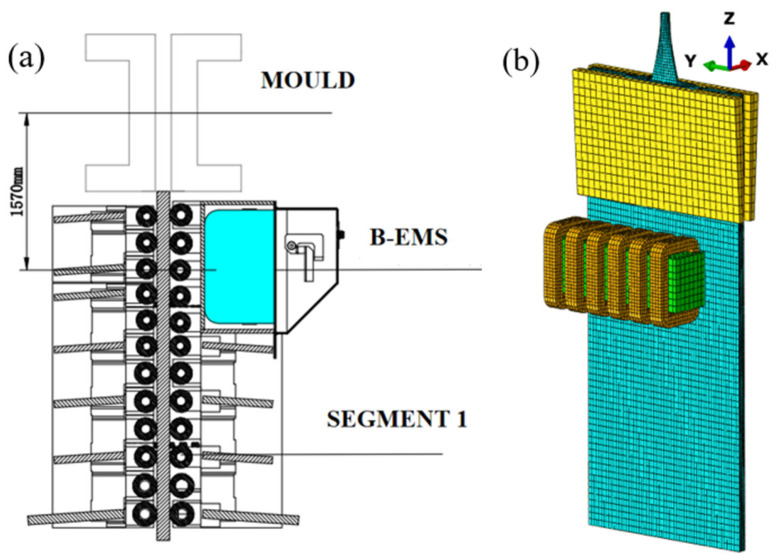
(**a**) Schematic of stirrer and installation on strand; (**b**) mesh structure of computational domain.

This experiment was performed using an operational vertical-bending type CSP continuous caster dedicated to silicon steel production at a domestic steel mill, which has a basic arc radius of 8 m and produces slabs with a cross-sectional dimension of 1680 mm × 72 mm. Figure 1a shows the specific installation location of the stirrer. The box-type electromagnetic stirrer (B-EMS) used in this study was installed outside the free rollers of a segment, also referred to as a roller-rear stirrer. Its installation form involves a single, independent setup on the inner side of the segment, differing from the paired installation of roller-type traveling wave stirrers. In addition, in contrast to conventional rotary electromagnetic stirring, B-EMS can exert a powerful directional electromagnetic force along the width of the casting billet, enabling it to better disrupt columnar grains and promote their conversion into equiaxed grains.

To simulate silicon steel continuous casting with high accuracy, a multiphysics coupled model was built according to the actual slab geometry of 1680 mm × 72 mm. This model achieves the coupling of electromagnetic, flow, temperature, and solidification fields. The model analyzes how varying B-EMS parameters affect the magnetic field and related transport processes in solidifying steel. The model uses the center of the meniscus as the origin and includes the coil, iron core, strand, and the air gap domain. The mesh consists of approximately 3 million cells, as shown in Figure 1b. The coordinate system is defined as follows: X-direction (narrow face), Y-direction (wide face), and Z-direction (casting direction).

### 2.1. Model Assumptions

(1) To simulate molten steel flow during steady speed casting, a low Reynolds number k-ε turbulence model is applied [22,23].

(2) The model treats the various thermophysical properties of the molten steel as constants, utilizes the Boussinesq approximation, and neglects factors such as chemical reactions, latent heat of phase transformation, and solidification shrinkage.

Constant thermophysical parameters ignore the influence on the heat transfer efficiency during the solidification process. The chemical reaction mainly affects the redistribution of solutes and the influence of chemical reaction products on thermophysical parameters. Ignoring solidification shrinkage means ignoring the gap formed between the slab and the mold during the slab solidification process, which will unpredictably affect the heat transfer on the slab surface. These assumptions mainly affect the simulation of the thermal field inside the entire slab. However, since the entire calculation domain is large, the influence of this assumption is relatively small, so an approximate simulation can be carried out.

(3) Regarding magnetohydrodynamic (MHD) coupling, based on the condition of a very low magnetic Reynolds number (Rm << 1), an assumption of one way coupling from the magnetic field to the flow was adopted, with time-averaged electromagnetic force used in the calculations.

(4) For geometric simplification, the model converted the curved secondary cooling zone into a vertical configuration to enhance computational and post-processing efficiency.

### 2.2. Governing Equations

#### 2.2.1. Electromagnetic Field

(1) $$\nabla \times \vec{E}=-\frac{\partial \vec{B}}{\partial t}$$

(2) $$\nabla \times \vec{B}=0$$

(3) $$\vec{J}=\nabla \times \frac{\vec{B}}{\mu }$$

(4) $$\vec{J}=\sigma (\vec{E})$$

where $\vec{Β}$—magnetic flux density (T), $\vec{Ε}$—electric field strength (V·m^−1^), $\vec{J}$—current density (A·m^−2^), μ—permeability (H·m^−1^), σ—conductivity (S·m^−1^).

(5) FE=12Re(j→×B→)

where $F_{E}$—time-averaged electromagnetic force (N·m^−3^); time-averaged electromagnetic force is the result of the vector cross-product of the induced current and the magnetic flux density. And $\vec{j}$—induced current density in steel melt (A·m^−2^); the complex current density expresses the numerical value of the induced current under the action of an alternating magnetic field and the phase change relative to the magnetic field [24,25,26].

#### 2.2.2. Flow and Solidification

(6) $$\frac{\partial (\rho u_{j})}{\partial x_{j}}=0$$

where ρ—density (kg·m^−3^), $u_{j}$—velocity component of the fluid in the $x_{j}$ direction.

(7) $$\rho \frac{\partial (u_{i}u_{j})}{\partial x_{j}}=-\frac{\partial (P)}{\partial x_{j}}+\frac{\partial }{\partial x_{j}}\left(\mu_{eff}\frac{\partial u_{i}}{\partial x_{j}}\right)+\frac{\partial }{\partial x_{i}}\left(\mu_{eff}\frac{\partial u_{j}}{\partial x_{i}}\right)+\rho g+F_{B}+F_{E}+S_{P}$$

where P—pressure (Pa), $\mu_{eff}$—effective viscosity coefficient (kg·(m·s)^−1^), calculated as:

(8) $$\mu_{eff}=\mu_{l}+\mu_{t}$$

where $\mu_{l}$—laminar viscosity (kg·m^−1^·s^−1^), $\mu_{t}$—turbulent viscosity (kg·m^−1^·s^−1^), calculated via the Kolmogorov–Prandtl relation [27,28]:

(9) $$\mu_{t}=\rho f_{\mu }C_{\mu }\frac{k^{2}}{\varepsilon }$$

The specific details of the low-Reynolds-number turbulence two-equation model can be found in the referenced literature [29,30]. $F_{E}$—electric field force; the electric field force is introduced into the continuous equation of molten steel, and the force caused by the temperature difference is also considered. $F_{B}$—thermal buoyancy:

(10) $$F_{B}=\rho g_{i}\beta (T-T_{l})$$

where β—thermal expansion coefficient (K^−1^), T—local temperature (K), $T_{l}$—liquidus temperature (K).

$S_{P}$—Darcy source term, representing the seepage rate in the mushy zone during solidification:

(11) $$S_{P}=\frac{{\left(1-f_{l}\right)}^{2}}{\left(f_{l}^{3}+\zeta \right)}A_{mush}(u-u_{p})$$

where $A_{mush}$—mushy zone constant (set to 5 × 108), ε is 0.0001; u—steel liquid flow rate (m·s^−1^), $u_{p}$—casting speed (m·s^−1^), $f_{l}$—liquid fraction:

(12) $$f_{l}=1-f_{s}=\left\{\begin{array}{cc} 1 & T\geq T_{l}\\ \frac{T-T_{s}}{T_{l}-T_{s}} & T_{s}<T<T_{l}\\ 0 & T\leq T_{s} \end{array}\right.$$

$T_{s}$ is the solidus temperature (K).


(13) $$\rho \frac{\partial (u_{i}H)}{\partial x_{j}}=\frac{\partial }{\partial x_{i}}\left(k_{eff}\frac{\partial T}{\partial x_{i}}\right)$$

The expression for the total system enthalpy H is:

(14) $$H=h_{ref}+\int_{T_{ref}}^{T}c_{p}dT+f_{l}L$$

where $k_{eff}$—effective thermal conductivity, $k_{eff}=k_{l}+\frac{c_{p}\mu_{t}}{{\mathrm{Pr}}_{t}}$, $c_{p}$—the specific heat capacity (J·(kg·K)^−1^), L—latent heat of solidification (J·kg^−1^), $h_{ref}$—reference enthalpy at the reference temperature $T_{ref}$.

#### 2.2.3. VOF Model

This study employs the VOF model to accurately simulate free surface fluctuations in the CSP thin slab mold, thereby tracking the steel slag interface. In the computational setup, only these two phases are considered for model simplification. During the simulation, the molten steel volume fraction is represented by αn, governed by the following equation for the nth phase:

(15) $$\frac{\partial \alpha_{n}}{\partial t}+U_{n}\cdot \nabla \alpha_{n}=0\;$$

For the secondary phase (liquid mold flux), the corresponding volume fraction is given by:

(16) $$\alpha_{1}=1-\alpha_{n}$$

Based on the value of αn in each computational cell, the cells can be categorized into three types: when αn = 1, the cell is entirely filled with molten steel and contains no mold flux; when αn = 0, it contains only liquid mold flux; when αn is between 0 and 1, the cell contains a mixture of molten steel and liquid mold flux.

### 2.3. Boundary Conditions

#### 2.3.1. Electromagnetic Field

(1) A six-coil B-EMS system is energized by three-phase alternating current, where each phase differs by 120°.
(2) The winding configuration is of the Cram type, uniformly wound along the axial direction of the iron core.
(3) This design necessitates two conditions: electrical insulation between the coil and core, and parallelism of the magnetic lines of force to the air region surface.

#### 2.3.2. Flow and Solidification

(1) Domain Inlet: A velocity inlet with velocity determined by casting speed and temperature fixed at casting temperature.
(2) Domain Outlet: The flow is considered fully developed, with zero gradients in the normal direction.
(3) Walls: Adiabatic and shear free conditions define the liquid film surface. On remaining walls, a no-slip condition applies, and empirical formulas determine the convective heat transfer coefficient from the cooling water flow rate.

### 2.4. Simulation Procedure

The numerical simulation method in this paper combines electromagnetic calculations with hydrodynamic calculations. To ensure complete electromagnetic effects and fully developed flow, the computational domain spans a strand section from the SEN inlet (0.5 m above meniscus) to 4 m below the meniscus. Electromagnetic field data are calculated and obtained by ANSOFT Maxwell 18.0 software (Ansys Inc., Canonsburg, PA, USA), which are then mapped into Fluent to solve the steady state flow, heat transfer, and solidification fields. The material composition (Table 1), along with key thermal properties and process parameters (Table 2), are detailed for the 50W600 silicon steel studied.

### 2.5. Model Validation

**Figure 2:**
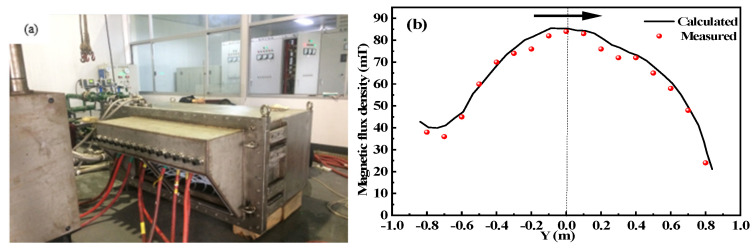
Distribution of magnetic flux density: (**a**) physical photo; (**b**) comparison between calculated and measured values of magnetic flux density.

Since B-EMS is installed behind the rear free roller, B-EMS often uses low-frequency power supply to reduce the eddy current on the free roller. Therefore, under the working condition of current 800 A and frequency 2 Hz (from industrial production experience), the magnetic flux density along the wide-face direction was experimentally measured at the slab center (X = 336 mm). The measured data were compared with simulation results, and the comparative distribution is shown in Figure 2. The magnetic flux density peaks at the center of the stirrer’s wide face and decreases gradually towards both sides, exhibiting a distinct single peak pattern. The close match between the measured values and the simulated curve validates the accuracy of the employed B-EMS electromagnetic model.

Due to the invisibility of the continuous casting process, it is impossible to verify the flow field and temperature field. Therefore, in the following part, we only analyze and discuss the action trend of B-EMS on molten steel during simulation, and make correspondence between the solidification structure of billet from industrial production and the analysis of numerical simulation.

## 3. Results Analysis and Discussion

### 3.1. Magnetic Field Simulation Analysis Under B-EMS Influence

**Figure 3:**
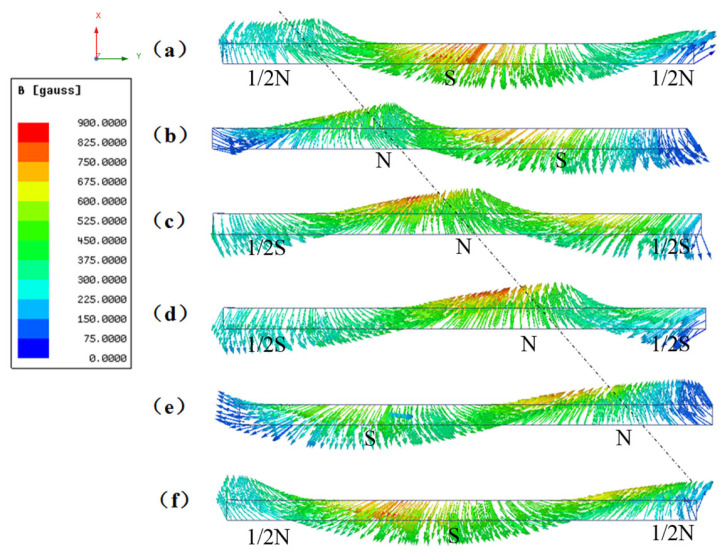
Magnetic induction intensity vector distribution map on the center cross-section (XY direction) of the billet: (**a**) 0 T, (**b**) 1/6 T, (**c**) 2/6 T, (**d**) 3/6 T, (**e**) 4/6 T, (**f**) 5/6 T.

Based on the rotating magnetic field principle, a box-type electromagnetic stirrer (B-EMS) is designed for application at the solidification front within the secondary cooling zone. The magnetic field distribution of this device is depicted in Figure 3. The vector diagram of the central XY plane clearly demonstrates the periodic variation of magnetic poles. Benefiting from the linear structure of the iron core, the magnetic flux density is capable of realizing traveling wave propagation. Specifically, magnetic lines of force emanate from the N pole, pass through the slab, and return via the S pole, thus forming an overall asymmetric distribution. This illustrates the working mechanism of this type of stirrer in the slab continuous casting secondary cooling zone, where it exerts a directional thrust on the molten steel via the traveling magnetic field. As the B-EMS is installed individually on the inner side of the segment, its magnetic field radiates along the X-direction, forming magnetic poles only beneath the slab. The magnetic field excited by the B-EMS exhibits only one pair of N-S poles at any given moment, which is attributed to its configuration of six coil windings generating a single pole-pair distribution. Within one cycle, the N and S poles move along the positive Y-direction. Consequently, the variation direction of the magnetic field excited by the B-EMS is oriented along the positive Y-axis.

**Figure 4:**
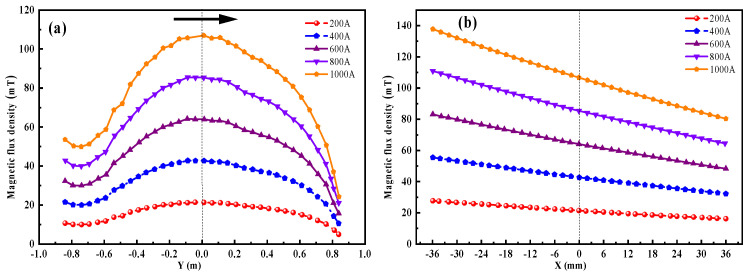
Distribution of effective magnetic flux density at the central plane of the stirrer (Z = −1.57 m): (**a**) X = 0.0 m line; (**b**) Y = 0.0 m line.

**Figure 5:**
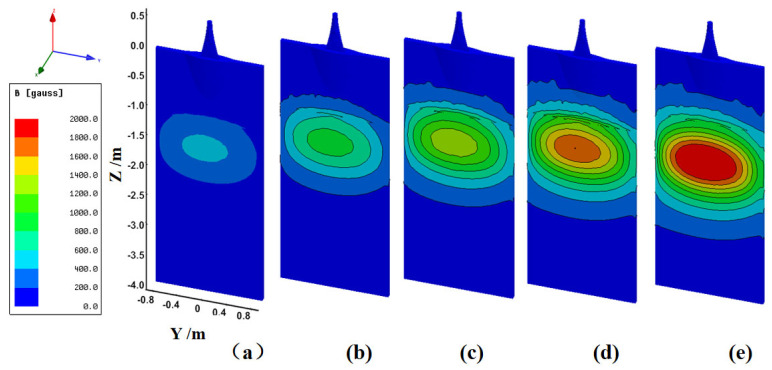
Magnetic flux density distribution on slab surface: (**a**) 200 A, (**b**) 400 A, (**c**) 600 A, (**d**) 800 A, (**e**) 1000 A.

Figure 4a and Figure 5 display the magnetic flux density distribution along the wide-face direction at the slab center under varying applied currents. The rising and falling trends of the magnetic flux density on the starting side and the pushing side are not symmetric, where the magnetic flux density at the pushing end is only 45% of that at the starting end. Results consistently show that the peak flux density occurs at the wide-side center. As the current increases incrementally from 200 A to 1000 A, the measured flux density at this location increases correspondingly to 21.3, 42.7, 64.2, 85.5, and 107.0 mT. This linear relationship between magnetic flux density and applied current aligns with fundamental electromagnetic theory, enabling straightforward prediction and control of magnetic field strength through current adjustment, thereby facilitating precise regulation of the stirring process. Figure 4b displays the B-EMS-induced magnetic flux density along the slab’s narrow-face centerline under various currents. As the current rises from 200 A to 1000 A, the magnetic field distribution across both sides of the slab shows a clear attenuation trend. Near the stirrer side, magnetic flux densities measure 137.7, 110.9, 85.2, 55.4, and 27.7 mT with increasing current. On the far side, the corresponding values are 80.3, 64.5, 48.3, 32.2, and 16.27 mT. The attenuation magnitude remains stable at approximately 58%. This consistent attenuation ratio across different currents suggests that, in addition to excitation intensity, the magnetic field penetration depth is also determined by slab geometry and material properties.

**Figure 6:**
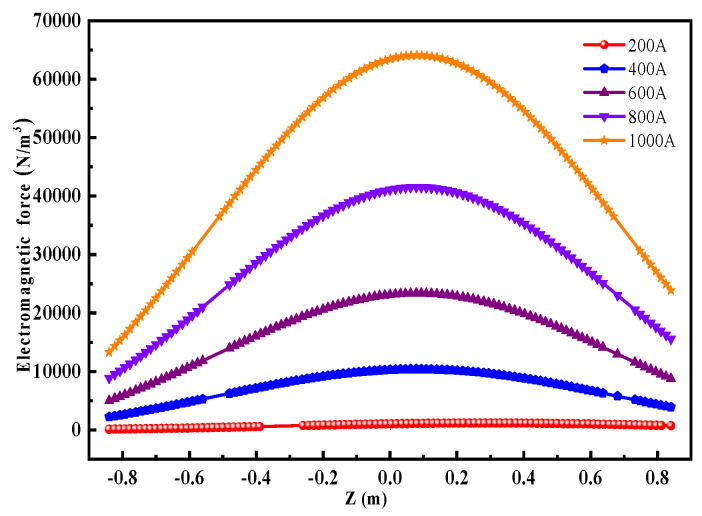
Distribution curve of electromagnetic force along the Y-axis direction.

**Figure 7:**
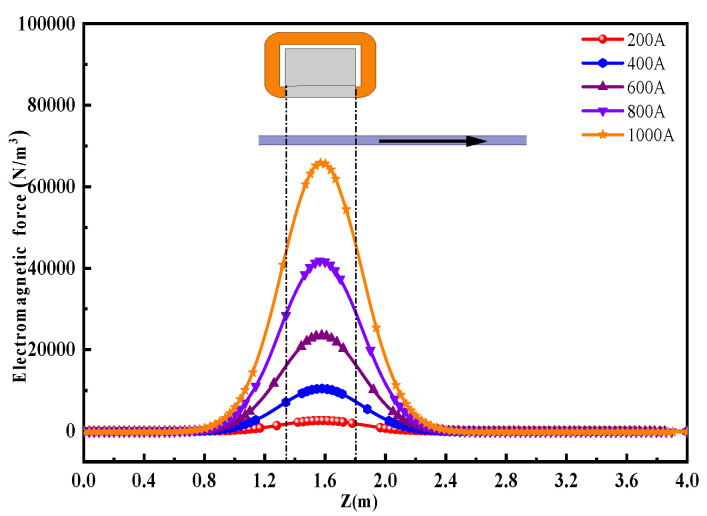
Distribution curve of the electromagnetic force along the line X = 0.0 m on the central plane (Y = 0.0 m) of the slab.

Influenced by the directional electromagnetic force of the traveling magnetic field, the slab exhibits variable B-EMS end effects, shown in Figure 6 [31]. Specifically, the electromagnetic force is stronger on the pushing side compared to the starting side, and its distribution trend does not follow the magnetic flux density variation in Figure 4a. A plausible explanation for this phenomenon is that the magnetic flux density on the pushing side experiences a more significant decrease, and such a substantial change in magnetic flux density induces a higher current density. Equation (5) indicates that magnetic flux density decreases and induced current density increases non-uniformly, with the latter change being more significant. This results in a stronger electromagnetic force on the pushing side. Figure 7 shows the electromagnetic force distribution along the casting direction exhibits a convex profile. Under different current conditions, the width of this convex curve remains stable between 0.8 m and 2.4 m, indicating that the effective action area of the magnetic field is independent of current intensity and is mainly concentrated in the region corresponding to the B-EMS iron core. As current increases from 200 A to 1000 A, the peak electromagnetic force density rises progressively to 2634, 10,419, 23,516, 41,792, and 65,864 N·m^−3^. This growth follows a quadratic relationship with the applied current. Combined with Formula (5), it can be seen that the electromagnetic force is the vector cross-product of the magnetic flux density and the induced current. The magnetic flux density is only a condition for the generation of the electromagnetic force, and it is the electromagnetic force that actually does work. Therefore, it is the electromagnetic force, rather than the magnetic flux density, that directly affects the scouring effect of molten steel on the solidification front [32,33,34].

Inside the slab, the magnetic field induced by the B-EMS possesses typical traveling magnetic field properties. Characterized by an overall asymmetric distribution, magnetic poles move continuously along the width direction (positive Y-axis) within one cycle. The electromagnetic force peaks at the wide-face center and varies with the square of the current. The electromagnetic force’s directional and periodic distribution in the slab is dictated by the structure and directional changes in the traveling magnetic field, thereby forming the basis for its effects on subsequent flow and solidification.

### 3.2. Flow Field Simulation Analysis Under B-EMS Influence

**Figure 8:**
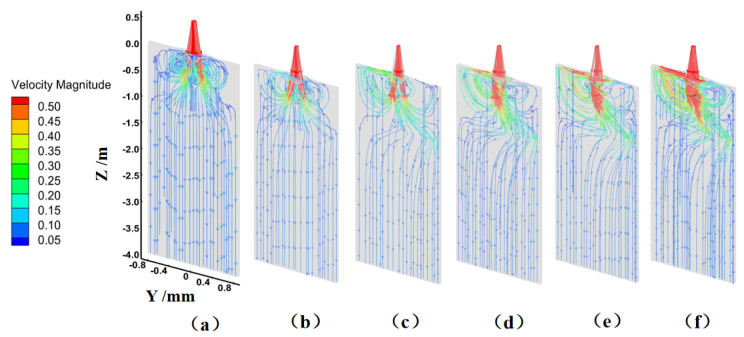
Flow field streamline diagram inside the slab: (**a**) 0 A, (**b**) 200 A, (**c**) 400 A, (**d**) 600 A, (**e**) 800 A, (**f**) 1000 A.

Within its effective range, the B-EMS markedly intensifies molten steel flow in the secondary cooling zone, as demonstrated by the velocity streamline distribution in Figure 8. Compared to the pushing side, the starting side of the mold exhibits more fully developed flow. This indicates that the electromagnetic force not only enhances molten steel flow within the mold but also extends its effective range. In the absence of B-EMS operation, part of the molten steel exiting the submerged entry nozzle (SEN) generates a recirculation flow within the mold, with a larger fraction entering the secondary cooling zone in the form of high-speed jets. Activating the B-EMS causes a distinct positive-Y deflection of the flow within its effective zone, aligning with the magnetic field’s phase variation. Increasing the current widens the affected range and amplifies the deflection. At a current of 200 A, the electromagnetic force is comparatively weak in magnitude. The flow field within the strand is only slightly disturbed, with streamlines showing minor irregularities. The overall trend remains similar to the case without B-EMS. When the current is increased to 400 A, the downward left jet from the SEN no longer appears. Instead, a large-scale recirculation flow is formed on the left lateral side of the slab. This is because the original downward left jet is subjected to a rightward electromagnetic thrust, which suppresses its flow. Meanwhile, the original downward right jet travels deeper and further under the action of the electromagnetic thrust. This reduces the recirculating flow towards the meniscus on the right-hand side of the mold cavity, allowing more molten steel to flow directly into the secondary cooling zone. Further increasing the current to 600 A and 800 A causes the molten steel flow to concentrate on the slab’s right side. Part of the downward flow recirculates back to the left side, while another part flows directly to the right, creating a clearly defined flow pattern throughout the entire transverse section. The observed steel flow trajectories indicate that currents within this range are relatively suitable for achieving the target flow modification.

In industrial production, the liquid surface flow velocity should not exceed 0.5 m/s, and the liquid surface fluctuation height should be within ±3 mm. However, increasing the current results in the emergence of increasingly larger high-velocity zones adjacent to the meniscus. At a current of 1000 A, the flow velocity exceeds 0.5 m/s across most of the meniscus area, indicating a significant enhancement in the driving capacity of the electromagnetic force for the molten steel. The flow jet on the left side of the mold exhibits a phenomenon similar to “impingement” or “blocking”. Excessive flow velocity can induce severe fluctuations at the meniscus, potentially leading to issues such as slag entrainment and argon bubble capture.

**Figure 9:**
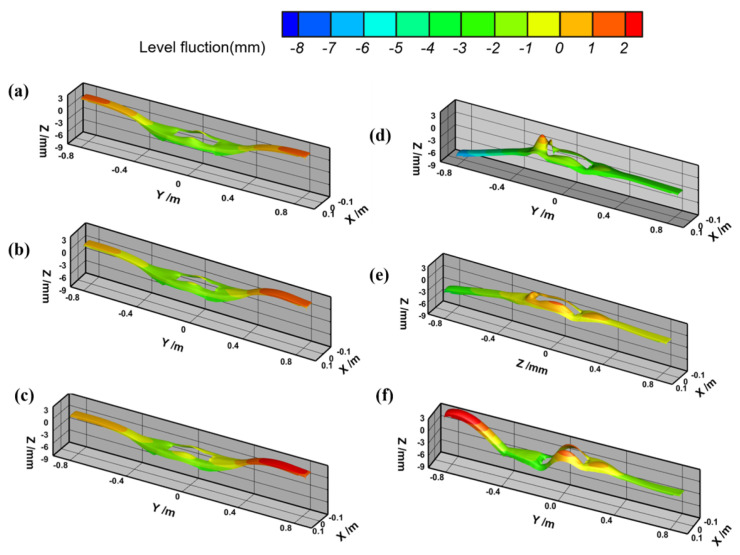
Fluctuation of the meniscus liquid level: (**a**) 0, (**b**) 200 A, (**c**) 400 A, (**d**) 600 A, (**e**) 800 A, (**f**) 1000 A.

Figure 9 illustrates the meniscus fluctuation under various excitation currents. It can be observed that without the B-EMS, the meniscus fluctuations on both sides of the mold are relatively large and symmetrical, exhibiting a depression in the center and protrusions on both sides. Maintaining a stable meniscus is crucial for the continuous casting process. Excessive fluctuation can lead to uneven growth of the primary solidified shell, resulting in surface cracks. It can also disrupt the lubrication stability of the mold flux, causing defects like slag entrainment, which severely impacts slab quality. When the current is 200 A, the meniscus fluctuation on the pushing side is higher, while the fluctuation on the starting side is suppressed. This trend becomes more pronounced at a current of 400 A. As the current is further increased to 600 A and 800 A, the fluctuation across the entire meniscus becomes relatively gentle. Not only is the fluctuation on the pushing side significantly suppressed, but the fluctuation pattern on the starting side also changes from a protrusion to a depression. This trend is consistent with the flow field characteristics analyzed in Figure 8. The leftward electromagnetic force pulls the upward recirculating flow on the left lateral side of the mold, thereby forming a meniscus shape that is depressed on the left and flatter on the right. When the current is increased to 1000 A, due to the formation of an upward recirculating flow on the left lateral side of the mold that impacts the end, the meniscus level on the starting side rises sharply. Therefore, while increasing the current initially suppresses meniscus fluctuation, an excessively high current can instead exacerbate it. Within the scope of this simulation study, a current of 800 A represents a condition where the B-EMS can both promote molten steel flow within the mold and suppress meniscus fluctuation.

**Figure 10:**
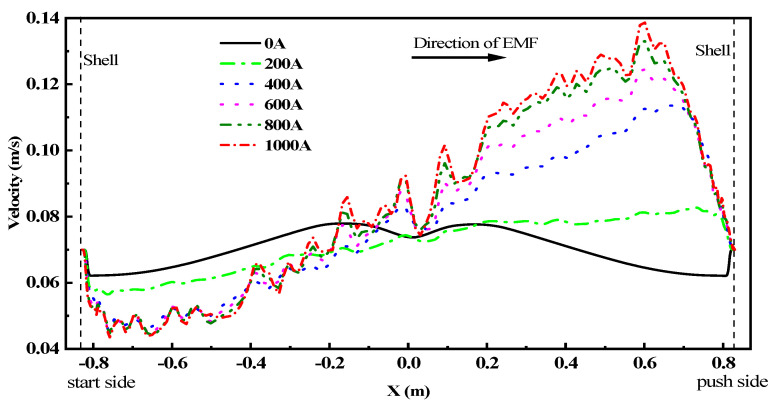
Velocity distribution at the stirrer center section under varying currents.

The velocity distribution along the centerline of the B-EMS effective zone under various currents is illustrated in Figure 10. Without a magnetic field, the molten steel velocity curves on the two wide faces are symmetrically distributed, with significant velocity gradients near the walls. The B-EMS induces a distinct flow velocity disparity between the starting and pushing sides, with the starting side exhibiting a lower overall velocity and a differing change trend. Furthermore, the velocity drop near the edge on the starting side is more pronounced compared to the case without B-EMS. Conversely, on the pushing side, the velocity trend is opposite: it is lower near the shell and gradually increases towards the interior.

When the current is 200 A, the velocity on the starting side decreases overall, while it increases slightly overall on the pushing side. The entire velocity profile exhibits an approximately centrosymmetric distribution. As the current increases from 400 A to 1000 A, the velocity magnitude and trend on the starting side remain relatively similar, with a minimum velocity reaching 0.043 m/s. In contrast, velocity on the pushing side rises markedly, with the magnitude of increase growing as the current is raised, reaching a maximum velocity of 0.138 m/s, which is three times that of the starting side.

Beyond altering the steel’s internal velocity distribution, the forced flow from the B-EMS also exerts a significant impact on the solidification front’s microstructure evolution. The strong transverse shear flow disturbs the growth conditions of columnar grains, making it difficult for them to maintain a stable, oriented growth direction, thereby weakening their preferential orientation. When the flow velocity reaches a certain intensity, the momentum impact and local shear stress generated by the flow field can fracture growing primary or secondary dendrite arms, causing them to detach and become free grains. These fragments can act as critical heterogeneous nucleation centers in undercooled molten steel, effectively triggering the prolific formation of equiaxed grains across the mushy region. The intensified molten steel flow enhances the exchange of heat and solutes at the interface of the solidification shell. This can lead to shell remelting on the pushed side and promote equiaxed grain formation, resulting in an uneven distribution of equiaxed grains across the slab cross-section.

### 3.3. Temperature Field Simulation Analysis Under B-EMS Influence

**Figure 11:**
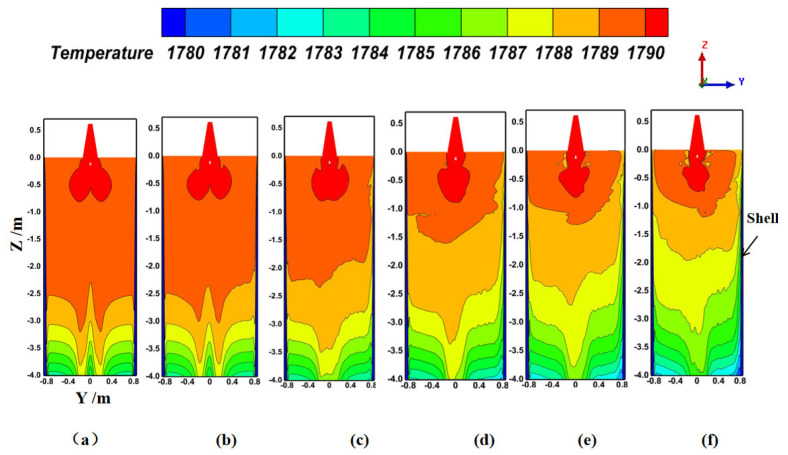
Temperature distribution along the casting direction at the slab width-wise center plane: (**a**) 0 A, (**b**) 200 A, (**c**) 400 A, (**d**) 600 A, (**e**) 800 A, (**f**) 1000 A.

Figure 11 illustrates the temperature distribution along the casting direction at the widthwise center plane of the slab. Without a magnetic field, the molten steel temperature across the slab width first decreases and then increases, showing a symmetric trend. This pattern is generally opposite to the velocity variation observed in Figure 8 without B-EMS, indicating that higher flow velocities lead to more efficient heat exchange and faster temperature drop. Without B-EMS, heat transfer across the entire fluid domain is insufficient. At approximately 2.5 m below the meniscus, the temperature in the central region still remains above 1789 K, while the temperature near the wall drops sharply to form a solidified shell. As the current increases, the temperature of the molten steel decreases significantly. When the current is 1000 A, the temperature of the molten steel drops to 1787 K.

After the application of B-EMS, heat transfer within the entire slab becomes more efficient. The temperature gradient throughout the casting direction is rendered more uniform, and the superheat in the core of the molten steel is notably lowered. Furthermore, heat transfer on the pushing side of the electromagnetic force is more uniform compared to the starting side. As analyzed earlier, the recirculating flow induced in the mold by the lower left flow jet accounts for this phenomenon. On the starting side, molten steel is supplied directly to the mold region by the SEN discharge, leading to a higher temperature compared to the pushing side. The promoting effect of B-EMS on heat transfer efficiency is positively correlated with the excitation current. More efficient heat transfer within the slab is achieved by higher currents, which enhance flow in the B-EMS zone and subsequently improve steel shell heat exchange and secondary cooling efficiency.

The application of electromagnetic stirring significantly adjusts the temperature field distribution within the strand, exerting a critical influence on the conditions for the columnar-to-equiaxed transition (CET). The forced convection induced by stirring accelerates heat transfer within the molten steel, substantially reducing the temperature gradient at the solidification front. According to the classical CET criterion proposed by Hunt [35,36], the transition occurs when the volume fraction of equiaxed grains exceeds a critical value, and this criterion can be expressed as a function of the temperature gradient GG, solidification velocity VV, and alloy composition. Specifically, a lower temperature gradient inhibits the stable growth of columnar dendrites by reducing the extent of the constitutionally undercooled zone ahead of the solid–liquid interface, causing them to lose their competitive growth advantage near the mushy zone. Concurrently, the rapid attenuation of superheat induced by electromagnetic stirring promotes the early formation of a large-volume undercooled zone, significantly increasing the nucleation rate. This provides the necessary thermodynamic driving force for the heterogeneous nucleation of equiaxed grains, effectively weakening the conditions that sustain continuous columnar growth.

### 3.4. Simulation Analysis of Shell Evolution Behavior Under B-EMS Influence

**Figure 12:**
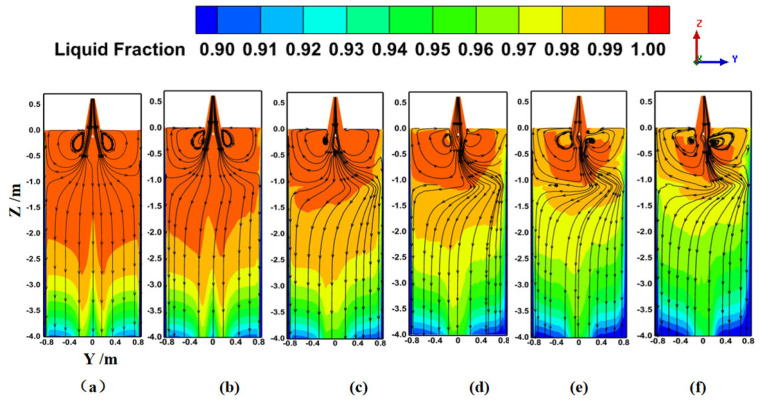
Liquid-phase fraction of XY center section of the casting billet: (**a**) 0 A, (**b**) 200 A, (**c**) 400 A, (**d**) 600 A, (**e**) 800 A, (**f**) 1000 A.

The solid–liquid phase distribution inside the slab further verifies that the B-EMS improves heat exchange efficiency across the secondary cooling region, with Figure 12 illustrating the liquid fraction distribution on the slab’s YZ cross-section. Electromagnetic thrust drives the directional flow of molten steel from the starting side toward the pushing side. This intensifies solidification interface heat transfer, promotes molten steel temperature uniformity, and provides favorable conditions for equiaxed grain formation.

Without B-EMS, a 0.99 liquid fraction core persists in the slab for about 2 m under the meniscus. As the B-EMS current increases, the liquid fraction on the pushing side of the electromagnetic force begins to decrease first, and a mushy zone coexisting with solid and liquid phases rapidly forms in the central region. Combined with the streamline distribution in Figure 10, it is evident that the electromagnetic force induces molten steel temperature homogenization. The liquid fraction within the entire slab decreases significantly with the increasing distortion of the streamlines (i.e., with increasing current). Uniform, effective scouring of the solidification front by molten steel enhances equiaxed grain formation potential. From a mechanistic perspective, the tangential flow of molten steel has a significant scouring effect on the dendrite front, causing the primary and secondary dendrite arms of columnar grains to break off and become free floating. This, in turn, promotes the generation of equiaxed grains at the solidification front. This is precisely the key theoretical basis for why high-thrust traveling wave electromagnetic stirring can inhibit columnar grain growth in slabs [36,37,38,39].

**Figure 13:**
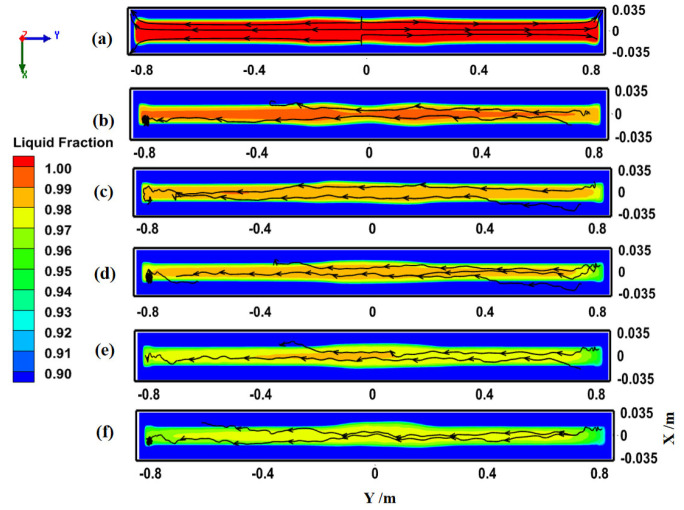
Liquid-phase fraction at the center section of the casting billet (Z = 4.0 m): (**a**) 0 A, (**b**) 200 A, (**c**) 400 A, (**d**) 600 A, (**e**) 800 A, (**f**) 1000 A.

Figure 13 presents contour plots of solid–liquid phase distribution and streamline plots of molten steel flow on a cross-section 4 m below the meniscus. From Figure 13a, it can be seen that without an electromagnetic field, the slab center is almost entirely in the liquid phase with a liquid fraction of 1, and the liquid phase occupies a large proportion. When the B-EMS is activated, the slab center transitions into a mushy zone of coexisting solid and liquid phases. Furthermore, a higher current leads to a lower liquid fraction and a smaller area of the mushy zone. This demonstrates that B-EMS improves heat exchange efficiency in both zones (mold and secondary cooling), reduces molten steel superheat, and optimizes solid–liquid phase uniformity. The B-EMS also influences the flow field trend at Z = 4 m, as shown in Figure 13a. In the absence of a magnetic field, flow on both slab sides is symmetrical, with molten steel flowing left and right respectively. When the B-EMS is active, the liquid phase within the slab flows towards the left side, opposite to the direction of the electromagnetic thrust. This can also be observed in Figure 13b–f. However, after the B-EMS pushes the molten steel towards the right side, the molten steel, upon contacting the slab shell, forms a flow trend towards the lower left, influencing the flow pattern throughout the entire slab.

**Figure 14:**
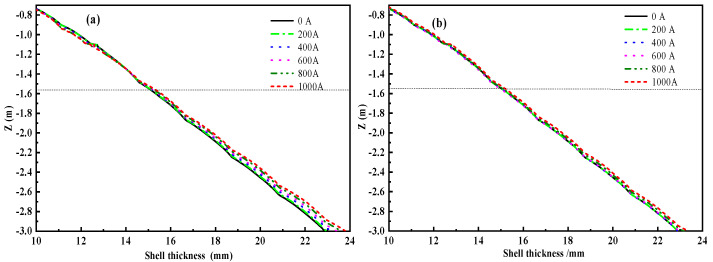
Shell thickness distribution at narrow-face center: (**a**) starting side, (**b**) pushing side.

As illustrated in Figure 14, B-EMS enhances the growth of the solidification shell on both narrow faces along the casting direction, with the shell thickness increasing as the current rises. In the absence of a magnetic field, the shell thickness at the starting side at Z = 3 m is 22.88 mm. As the current increases from 200 A to 1000 A, the corresponding shell thicknesses are 22.98 mm, 23.2 mm, 23.41 mm, 23.62 mm, and 23.82 mm, respectively. On the pushing side, the shell thickness increases from 22.89 mm without a magnetic field to 23.4 mm under 1000 A excitation, with intermediate values of 22.91 mm, 22.96 mm, 23.03 mm, and 23.21 mm at 200 A, 400 A, 600 A, and 800 A, respectively. When the B-EMS current exceeds 600 A, the shell thickness on both the starting and pushing sides reaches above 23 mm. The increase in shell thickness originates from the enhanced molten steel flow velocity induced by B-EMS, which intensifies heat transfer within the interior and at the solidification front, thereby promoting the solidification process.

Meanwhile, it is observed that after B-EMS application, even under stable casting conditions, the shell thickness on the pushing side is consistently slightly thinner than that on the starting side. This unique phenomenon cannot be explained solely by conventional principles of molten steel flow velocity and heat transfer behavior; it likely involves the coupled effects of multiple mechanisms, including electromagnetic force-driven asymmetric flow, solute redistribution, and dendrite fragment migration.

From the perspective of flow field distribution, the traveling magnetic field generates a directional electromagnetic thrust across the slab width, driving molten steel flow from the starting side toward the pushing side. This directional flow causes the solidification front on the pushing side to be continuously scoured by high-temperature molten steel, with enhanced thermal convection increasing local heat flux, thereby delaying shell growth on this side. In contrast, on the starting side, part of the fluid kinetic energy is converted into pressure energy at the solidification front, resulting in relatively weaker convective heat transfer, which favors shell thickening. From the perspective of solute transport, the electromagnetic thrust drives solute-enriched molten steel from the solidification front on the starting side toward the pushing side. According to solute redistribution theory, solute enrichment leads to a decrease in the liquidus temperature, forming a constitutionally undercooled zone. As the pushing side continuously receives solute-enriched molten steel, the liquidus temperature at its solidification front decreases, correspondingly lowering the actual solidification temperature, thereby delaying the solidification process. From the perspective of quantitative heat distribution analysis, the shell thickness difference between the starting and pushing sides exhibits a trend of initial expansion followed by stabilization as the current increases. In the 200–600 A range, the thickness difference gradually expands from 0.07 mm to 0.38 mm; when the current exceeds 600 A, the difference stabilizes at approximately 0.4–0.5 mm. This may indicate the existence of a critical flow velocity threshold, beyond which the transport effect of electromagnetic thrust on molten steel tends to saturate, and the difference in solidification conditions between the two sides reaches a dynamic equilibrium.

In summary, the shell thickness difference between the starting and pushing sides results from the combined effects of electromagnetic force-driven asymmetric flow, solute redistribution, and dendrite fragment migration. This phenomenon not only reveals the complexity of solidification behavior under B-EMS but also provides important insights for optimizing stirrer installation positions and process parameters.

### 3.5. Macroscopic Microstructure Analysis Under B-EMS Influence

**Figure 15:**
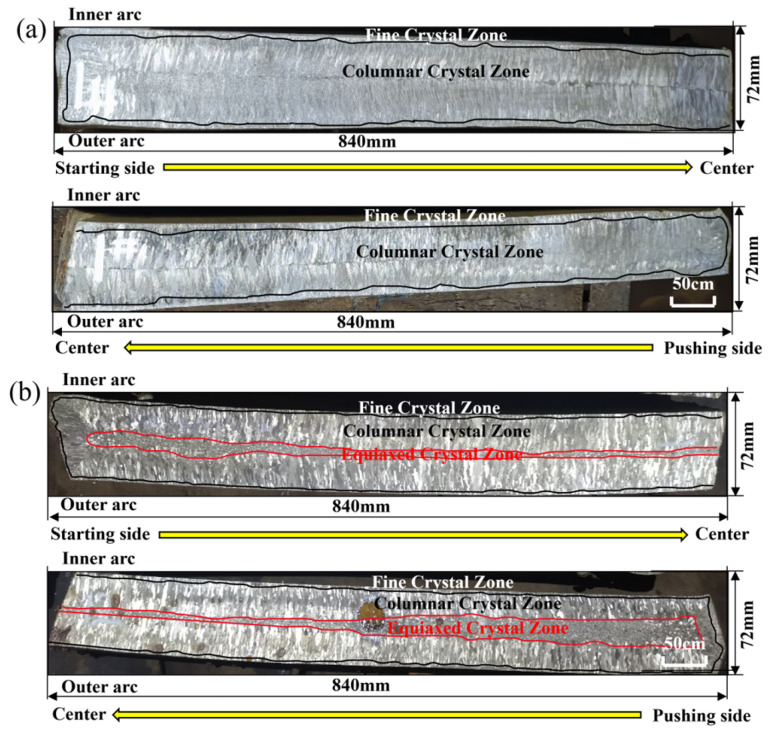
Non-oriented silicon steel W20PD slab macrostructure: (**a**) without B-EMS, (**b**) with B-EMS (800 A).

Based on the preceding analysis, a current of 800 A yielded the optimal overall performance in terms of meniscus stability, flow field, and temperature field. Nevertheless, the step span of 200 A leads to less refined conclusions, and 800 A may not be the optimal current parameter. On balance, 800 A is a relatively better electromagnetic parameter. Consequently, continuous casting experiments were conducted on the silicon steel slab caster under two conditions: without electromagnetic stirring and with B-EMS applied at 800 A. Figure 15 presents the macrostructure of the slab cross-section. For both conditions, rapid nucleation occurs during the initial solidification stage due to intense external cooling, forming a chill layer on the slab surface. The high cooling rate and steep temperature gradient from the shell to the mushy zone and liquid core drove the sustained growth of columnar grains, which extend from the shell toward the center of the liquid pool.

Without B-EMS, a sharp through-thickness temperature gradient was maintained throughout the solidification process. As a result, the as-cast structure of the silicon steel slab was dominated by well-developed columnar grains, growing perpendicular to the wide face of the slab. This columnar-dominated as-cast structure, due to its strong anisotropy, is a primary cause of internal quality issues in subsequent processing. With B-EMS applied, the slab presented a distinct three-zone structure: a chill layer, a columnar grain zone, and an equiaxed grain zone. A well-defined equiaxed grain zone formed at the slab center, with the equiaxed grain ratio rising from 0% to 30%.

This improvement is attributed to the shear force generated by electromagnetically driven molten steel flow, which fragments growing columnar dendrites. These fragments transform into free grains and serve as nucleation sites, promoting the formation of equiaxed grains during subsequent solidification stages. Additionally, the reduction in molten steel superheat favors the survival of equiaxed grains, thereby inhibiting columnar grain growth.

Notably, the macrostructure reveals a significant asymmetry in the distribution of equiaxed grains across the slab width—the equiaxed grain ratio on the pushing side is slightly higher than that on the starting side. As established previously, the difference in electromagnetic force leads to asymmetric flow fields on both sides. Dendrite fragments generated by flow-induced shear forces are transported from the starting side to the pushing side, making the pushing side an “enrichment zone” for dendrite fragments and increasing the heterogeneous nucleation rate on this side. However, the pushing side is continuously scoured by high-temperature molten steel, resulting in locally higher temperatures that are unfavorable for equiaxed grain survival. Under these conditions, the enriched fragments must dissipate heat through remelting to compensate for the temperature gradient effects. Ultimately, this results in the pushing side exhibiting a slightly higher equiaxed grain ratio than the starting side.

Thus, the macrostructure observations show good agreement with numerical simulations, validating the reliability of the established model from multiple perspectives and providing a theoretical foundation for parameter optimization in industrial applications.

## 4. Conclusions

Through numerical simulations and industrial experiments, the electromagnetic characteristics of the box-type electromagnetic stirrer (B-EMS) and its effects on solidification, heat transfer, and flow behavior during silicon steel continuous casting were investigated. Conclusions are as follows:

(1) B-EMS significantly modifies the molten steel flow pattern. At 200–400 A, upward recirculating flow on the starting side suppresses meniscus fluctuations; at 600–800 A, the flow redistributes uniformly across the wide face while maintaining meniscus stability. However, at 1000 A, excessive flow causes a sharp rise in the meniscus level on the starting side, increasing the risks of slag entrainment and surface cracks.
(2) The forced convection induced by B-EMS enhances heat transfer within the strand, rapidly reducing superheat and homogenizing temperature distribution. At 800 A, the shell thickness at the narrow-face center increases from 22.9 mm to 23.6 mm on the starting side and 23.2 mm on the pushing side.
(3) B-EMS promotes equiaxed grain formation by reducing superheat and providing strong shear forces, supplying numerous free grains for equiaxed grain growth. Industrial trials under 800 A conditions confirmed that the equiaxed grain ratio reaches approximately 30%, effectively mitigating quality defects in subsequent processing caused by material anisotropy.

Despite these findings, several limitations of this study should be acknowledged, which also point to promising directions for future research. First, although the established multiphysics model effectively captures the macroscopic distributions of electromagnetic, flow, and temperature fields, it does not fully resolve the complex two-phase flow behavior within the mushy zone. Future work could incorporate multi-phase models or porous media theory to more accurately describe transport phenomena in this region. Second, while dendrite fragmentation is identified as a key mechanism for equiaxed grain nucleation, the quantitative relationships among fragment generation, transport, survival probability, and flow intensity remain unclear. Combining phase field simulations with macroscopic models would enable cross-scale coupling from fluid flow to microstructural evolution. Third, this study focused mainly on current intensity effects, while other critical parameters—including current frequency and stirrer installation position—deserve systematic investigation. Furthermore, the conclusions drawn from silicon steel require validation across other steel grades to establish universal principles governing B-EMS effects.

## Figures and Tables

**Table 1:** Chemical composition of 50W600 silicon steel (mass fraction, %).

C	Als	Mn	P	S	Si
≤0.003	0.2~0.5	0.2~0.6	≤0.025	≤0.005	1.2~1.6

**Table 2:** Steel thermophysical properties and continuous casting parameters.

Parameters	Value	Parameters	Value
Slab cross-section	1680 × 72 mm^2^	Liquidus temperature	1768 K
Distance to meniscus of B-EMS	1.57 m	Solidus temperature	1703 K
Relative permeability of each material	1	Specific heat	720 (kg·K)^−1^
Relative permeability of iron core	1000	Latent heat of solidification	272,000 J·kg^−1^
Conductivity of molten steel	7.14 × 10^5^ S·m^−1^	Superheat degree	20 °C
Specific water flow	0.4 L·kg^−1^	Molten steel density	7200 kg·m^−3^
Casting speed	3.5 m·min^−1^	Molten steel viscosity	0.0055 kg·(m·s)^−1^
Inlet velocity	1.4 m·s^−1^	Thermal conductivity of molten steel	32 W·(m·K)^−1^
Inlet temperature	1790 K	Heat flux in the mold	2.7 × 10^6^ W·m^−2^
		Heat transfer coefficient in the secondary cooling zone	800 W·(m^2^ K)^−1^

## Data Availability

The original contributions presented in this study are included in the article. Further inquiries can be directed to the corresponding author.
